# Recurrent Syncope Attributed to Left Main Coronary Artery Severe Stenosis

**DOI:** 10.1155/2015/782347

**Published:** 2015-01-15

**Authors:** Min Li, Xinyi Zheng, Hua Liu, Yujie Liu

**Affiliations:** ^1^Capital Medical University, Beijing 100069, China; ^2^Tianjin Medical University, Tianjin 300070, China; ^3^2nd Department of Cardiology, Cangzhou Central Hospital, Hebei 061001, China; ^4^4th Department of Cardiology, Tianjin Chest Hospital, Tianjin 300051, China

## Abstract

Patients with acute coronary syndrome (ACS) rarely manifest as recurrent syncope due to malignant ventricular arrhythmia. We report a case of a 56-year-old Chinese male with complaints of paroxysmal chest burning sensation and distress for 2 weeks as well as loss of consciousness for 3 days. The electrocardiogram (ECG) revealed paroxysmal multimorphologic ventricular tachycardia during attack and normal heart rhythm during intervals. Coronary angiograph showed 90% stenosis in left main coronary artery and 80% stenosis in anterior descending artery. Two stents sized 4.0∗18 mm and 2.75∗18 mm were placed at left main coronary artery and anterior descending artery, respectively, during percutaneous coronary intervention (PCI). The patient was discharged and never had ventricular arrhythmia again during a 3-month follow-up since the PCI. This indicated that ventricular tachycardia was correlated with persistent severe myocardial ischemia. Coronary vasospasm was highly suspected to be the reason of the sudden attack and acute exacerbation. PCI is recommended in patients with both severe coronary artery stenosis and ventricular arrhythmia. Removing myocardial ischemia may stop or relieve ventricular arrhythmia and prevent cardiac arrest.

## 1. Introduction

ACS is mainly characterized by acute myocardial infarction and unstable angina pectoris which usually manifest as chest pain or distress, shortness of breath, or radiating pain such as pharyngalgia or toothache. But ACS patients rarely manifest recurrent syncope due to malignant arrhythmia. We report a case of paroxysmal multimorphologic ventricular tachycardia caused by left main coronary artery severe stenosis.

## 2. Case Report

**Figure 1:**
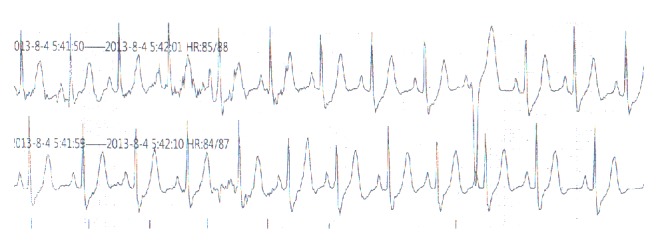
ECG record of 1 minute before attack at 5:41 a.m. on August 4, 2013.

**Figure 2 fig2:**
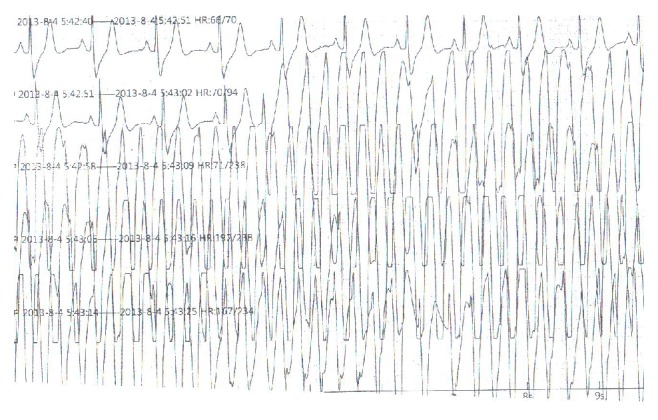
ECG record during attack at 5:42 a.m. on August 4, 2013.

**Figure 3 fig3:**
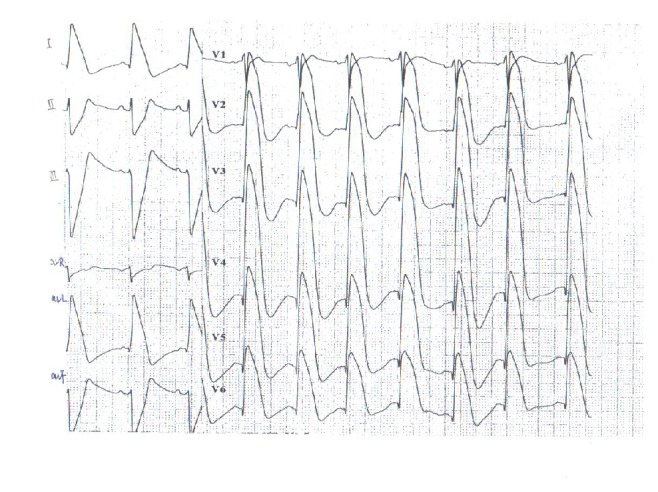
ECG record of 8 minutes after attack at 5:50 a.m. on August 4, 2013.

**Figure 4 fig4:**
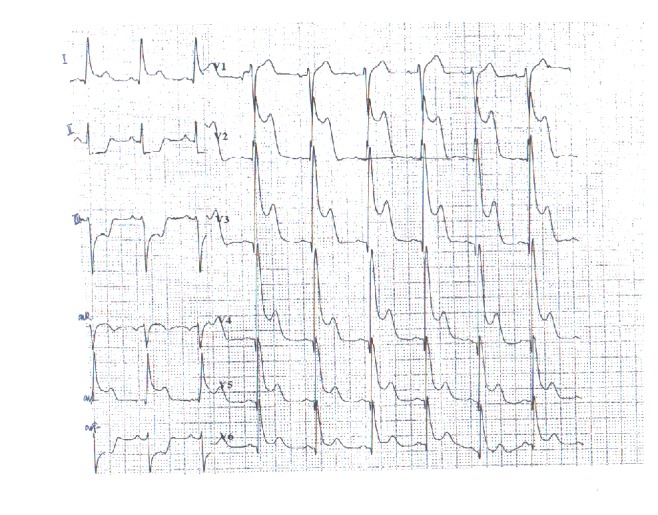
ECG record of 16 minutes after attack at 5:58 a.m. on August 4, 2013.

The patient was a 56-year-old Chinese male who was admitted to Tianjin Chest Hospital on August 4, 2013, with complaints of paroxysmal chest burning sensation and distress for 2 weeks and loss of consciousness for 3 days. Two weeks before admission, he had a chest burning sensation, distress, and toothache for a duration of 2-3 minutes while riding a bicycle. He had no vomiting, nausea, dizziness, or palpitation. He suffered from a chest burning sensation and distress again followed by loss of consciousness and faint 3 days prior to admission in the bathroom. The patient resumed consciousness several minutes later without abnormal movement of extremities and aphasia. The symptoms described above occurred after a noontime nap 2 days before his admission. He was sent to local hospital the same day. Aspirin, isosorbide mononitrate, and antilipemic agent (unknown) were given by local doctors. Electrocardiogram and skull CT revealed no abnormality. Emergency treatments including cardioversion and intravenous lidocaine were performed after the patient had loss of consciousness with upgazing eyes, tics of limbs, and urinary incontinence during admission of the following morning. The patient resumed consciousness several minutes later and the ECG returned to normal half an hour later. The ECG before, during, and after attack was recorded as in Figures 1–5.

**Figure 6:**
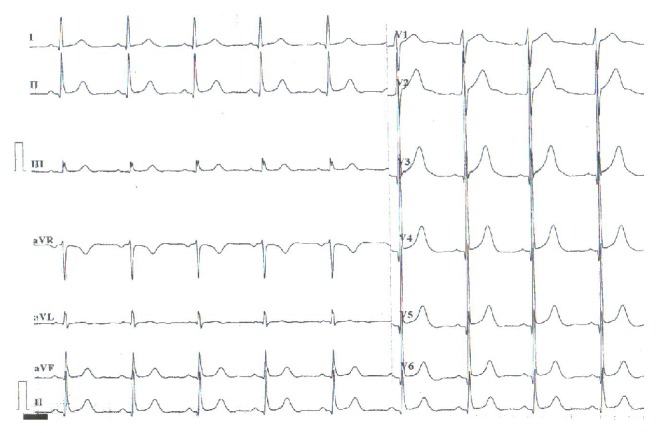
ECG at admission at 9:30 a.m. on August 4, 2013.

Thereafter, the patient was transferred to our hospital on August 4, 2013. ECG at the admission showed no severe abnormality and was recorded as shown in Figure 6.

*Past History.* The patient had hypertension up to 160/110 mmHg for 3-4 years and did not take antihypertensive medications regularly.

*Physical Examination.* Active position with consciousness, body temperature 36.5°C, blood pressure 125/80 mmHg, pulse rate 70 bpm, heart rate 70 bpm, respiratory rate 16/minute. The patient had regular heart rhythm, strong heart sound, clear breathing sounds in both lung fields, soft and flat abdomen, no murmur at each valvular area, and no edema in ankles. Border of cardiac dullness was not enlarged.

Laboratory data revealed high TSH of 5.37 *μ*IU/mL, high neutrophil percentage of 80.61%, and low lymphocyte percentage of 13.12%. Glucose, triacylglycerol, total cholesterol, LDL-C, VLDL-C, HDL-C, CK, CK-MB, TnI, and BNP levels were within normal ranges.

Echocardiogram showed aortic arteriosclerosis, segmental abnormal movement of left ventricular wall, and ejection fraction of 68%. Chest X-ray showed dilated aorta, heart chest ratio 0.48, and clear pulmonary field.

The patient was given aspirin, clopidogrel, isosorbide mononitrate, diltiazem, atorvastatin, metoprolol, and intravenous amiodaronum for treatment after admission. However, symptoms as chest distress, palpitation, and loss of consciousness occurred twice on the 3rd day after the admission. ECG monitoring recorded ventricular tachycardia.

Ventricular tachycardia vanished after cardioversion was performed. ST segment was arched upward and T wave was inversed on V1-V6 lead. ST-segment depression was also found on II, III, and avF lead. ECG gradually returned to normal after approximately 10 minutes. Waveform on ECG was very similar to previous attacks as recorded above.

**Figure 7:**
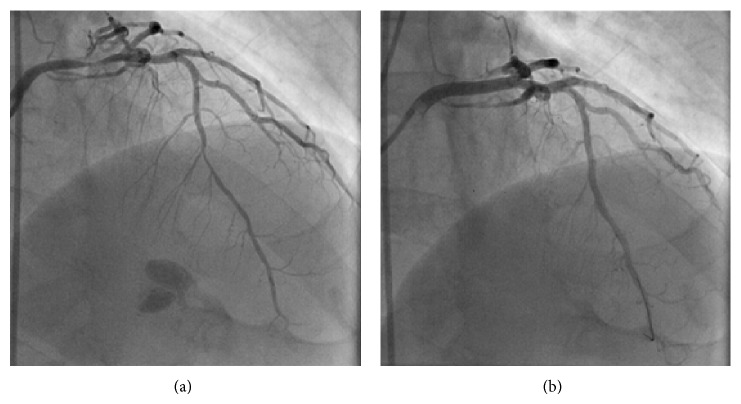
(a) The left graph shows the coronary angiograph before PCI. (b) The right-hand map showed coronary angiograph after PCI.

Coronary angiograph on the 5th day of the admission revealed 90% stenosis in left main coronary artery and 80% stenosis in anterior descending artery (Figure 7(a)). The left circumflex artery and right coronary artery revealed no abnormality. Two stents sized 4.0 ∗ 18 mm and 2.75 ∗ 18 mm (XIENCE V) were placed in left main coronary artery and anterior descending artery, respectively, during PCI. Coronary angiograph after PCI revealed effective filling of coronary artery (Figure 7(b)).

The patient recovered and was discharged on the 9th day after admission. He never had the symptoms again since the coronary artery intervention during 3-month follow-up. The patient was given aspirin, clopidogrel, isosorbide mononitrate, diltiazem, and atorvastatin for treatment after discharge.

## 3. Discussion

The patient had a recurrent chest burning sensation, distress, and loss of consciousness. ECG during the attack revealed paroxysmal multimorphologic ventricular tachycardia followed by arched ST segment and inversed T wave. These symptoms could be due to the following causes.

### 3.1. ACS

The patient had typical ACS symptoms such as chest burning pain, chest distress, and toothache during labor and shares the risk factors of ACS such as male gender, smoking, hypertension, and age above 45 years. The symptoms appear with higher frequency in the following 3 days. ST elevations after the attack also provide evidence supporting severe and extensive myocardial ischemia due to unstable plaques. Coronary angiograph demonstrated severe stenosis at left main coronary artery and anterior descending artery. Coronary blood flow can be obviously affected under stenosis ≥50%. 90% stenosis in left main coronary artery in this case indicated a strong correlation between the stenosis and the extensive transmural ischemia. It is also reported in another study that extensive transmural ischemia could cause ventricular arrhythmia [1]. All the evidences supported that multimorphologic ventricular tachycardia was correlated with myocardial ischemia. The vasodilator administered after admission cannot reverse the stenosis and did not stop the episode. PCI was proved effective in existence of severe coronary stenosis. No recurrence emerged after PCI during the follow-up period.

### 3.2. Coronary Vasospasm

Normal levels of myocardial enzymes indicated variant angina. The quiescent attacks were irrelevant to exercises. Coronary vasospasm was highly suspected to be the reason of the sudden attack and acute exacerbation. Recurrent syncope seemed to correlate with temporary reduction of coronary blood supply due to coronary vasospasm. Vasospasm tends to occur near atherosclerotic plaques in proximal coronary artery occlusion [2]. This may be due to increased contractility of ischemic vascular wall, endothelium injury, high concentration of local vasoconstrictors, high responsiveness of vascular smooth muscle to vasoconstrictors, and rupture of unstable atherosclerotic plaques [3]. However, ergonovine provocative test was not performed before PCI for the safety of the patient; vasospasm at left main coronary artery with 90% stenosis may induce ventricular fibrillation or even cardiac arrest. Intracoronary nitroglycerin was regularly used before PCI. Thus, it would not be helpful to perform ergonovine test after PCI to assess the effectiveness of the therapy due to the interference by intracoronary nitroglycerin.

## 4. Conclusion

Ventricular tachycardia may end up with ventricular fibrillation which could cause death. Temporary ECG changes caused by myocardial ischemia are usually difficult to capture. Thereafter, a 24 h holter and ECG monitoring may help in diagnosing. Antiarrhythmic medications such as amiodarone alone or even in combination with coronary vasodilators such as calcium channel blocker (CCB) and nitrate medicine cannot terminate ventricular arrhythmia while coronary artery stenosis remains severe and myocardial ischemia still exists. Therefore, PCI is recommended as first choice in patients with both severe coronary artery stenosis and ventricular arrhythmia. Removing myocardial ischemia correlated with coronary artery stenosis may eventually stop or at least relieve ventricular arrhythmia and prevent cardiac arrest. PCI in the case obtained a satisfactory result.

## Figures and Tables

**Figure 5:**
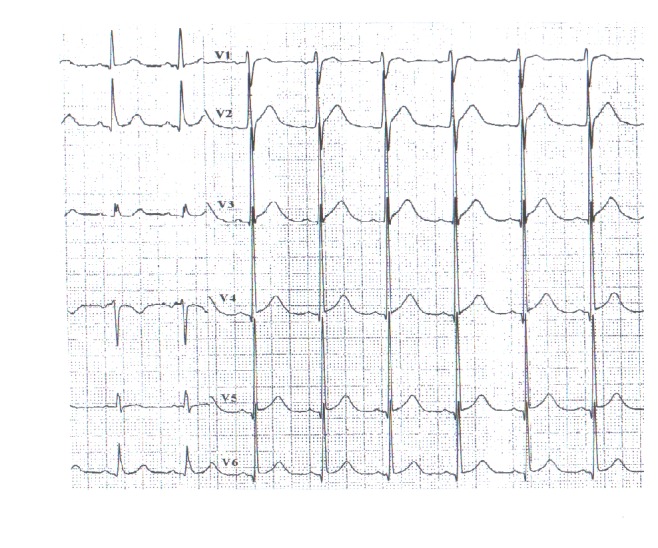
ECG record of 32 minutes after attack at 6:14 a.m. on August 4, 2013.
